# Using PBPK modeling to supplement clinical data and support the safe and effective use of dolutegravir in pregnant and lactating women

**DOI:** 10.1002/psp4.13251

**Published:** 2024-10-30

**Authors:** Jia Ning, Amita Pansari, Karen Rowland Yeo, Aki T. Heikkinen, Catriona Waitt, Lisa M. Almond

**Affiliations:** ^1^ Certara Predictive Technologies Division Sheffield UK; ^2^ Department of Pharmacology and Therapeutics University of Liverpool Liverpool UK; ^3^ Infectious Disease Institute Makerere University College of Health Sciences Kampala Uganda; ^4^ Royal Liverpool University Hospital Liverpool UK

## Abstract

Optimal dosing in pregnant and lactating women requires an understanding of the pharmacokinetics in the mother, fetus, and breastfed infant. Physiologically‐based pharmacokinetic (PBPK) modeling can be used to simulate untested scenarios and hence supplement clinical data to support dosing decisions. A PBPK model for the antiretroviral dolutegravir (mainly metabolized by UGT1A1) was verified using reported exposures in non‐pregnant healthy volunteers, pregnant women, and the umbilical cord, lactating mothers, and breastfed neonates. The model was then applied to predict the impact of UGT1A1 phenotypes in extensive (EM), poor (PM), and ultra‐rapid metabolizers (UM). The predicted dolutegravir maternal plasma and umbilical cord AUC in UGT1A1 PMs was 1.6‐fold higher than in EMs. The predicted dolutegravir maternal plasma and umbilical cord AUC in UGT1A1 UMs mothers was 1.3‐fold lower than in EMs. The predicted mean systemic and umbilical vein concentrations were in excess of the dolutegravir IC_90_ at 17, 28, and 40 gestational weeks, regardless of UGT1A1 phenotype, indicating that the standard dose of dolutegravir (50 mg q.d., fed state) is generally appropriate in late pregnancy, across UGT1A1 phenotypes. Applying the model in breastfed infants, a 1.5‐, 1.7‐, and 2.2‐fold higher exposure in 2‐day‐old neonates, 10‐day‐old neonates, and infants who were UGT1A1 PMs, respectively, compared with EMs of the same age. However, it should be noted that the exposure in breastfed infants who were UGT1A1 PMs was still an order of magnitude lower than maternal exposure with a relative infant daily dose of <2%, suggesting safe use of dolutegravir in breastfeeding women.

**Study Highlights**

**WHAT IS THE CURRENT KNOWLEDGE ON THE TOPIC?**

Optimal dosing in pregnant and lactating women requires an understanding of the pharmacokinetics of the mother and their babies. The PBPK model can be used to simulate untested scenarios and hence supplement clinical data to support dosing decisions in studied populations.

**WHAT QUESTION DID THIS STUDY ADDRESS?**

After demonstrating the recovery of observed exposure in non‐pregnant healthy volunteers, pregnant, umbilical cord, lactating mothers, and their breastfed neonates, the PBPK model was then applied to predict the dolutegravir exposure in clinically untested scenarios, namely, different combinations of maternal and neonate/infant UGT1A1 phenotypes to support safe and effective use of dolutegravir in mothers living with HIV.

**WHAT DOES THIS STUDY ADD TO OUR KNOWLEDGE?**

Our simulations indicate that the standard dose of dolutegravir (50 mg q.d.) in a fed state is generally appropriate in pregnant and lactating women, regardless of the phenotype of UGT1A1.

**HOW MIGHT THIS CHANGE DRUG DISCOVERY, DEVELOPMENT, AND/OR THERAPEUTICS?**

PBPK modeling can be used to supplement clinical data in pregnancy and lactation to assess the interplay between covariates in populations that are difficult to study.

## INTRODUCTION

Mother‐to‐child transmission (MTCT) of HIV can occur during pregnancy, delivery, or after birth during breastfeeding.^1^ In 2022, there were 1.2 million pregnant women globally living with HIV, of whom ~82% received antiretroviral therapy (ART) to prevent MTCT.^2^ Universal access to antiretroviral therapy means that all people, including women diagnosed with HIV during pregnancy and breastfeeding, should be initiated on lifelong antiretroviral therapy. In view of this, and the low rates of MTCT of HIV with suppressive ART,^3^ the WHO infant feeding guidelines were updated in 2016 to recommend exclusive breastfeeding for 6 months followed by complementary feeding for up to 2 years^4^; previous caution against mixed feeding or prolonged breastfeeding which were based on evidence from the pre‐ART era were removed. Guidelines from high‐income countries do not actively recommend breastfeeding, but where a mother is virologically suppressed and adherent to ART, she should be supported in her choice. However, many questions remain about the exact rate of MTCT, the role of cell‐associated virus in transmission when plasma viral load is fully suppressed, the extent and impact of drug transfer to the breastfed infant; and the lack of evidence‐based information to answer these.^5^ Pregnant and breastfeeding women have been systematically excluded from many clinical trials of newer ART, although this perspective is increasingly being challenged.^6^ It is important to understand drug exposure in pregnancy and breastfeeding because lower concentrations might cause therapeutic failure, and therefore increase the risk of MTCT, whereas excessive exposure might risk toxicity. Subtherapeutic concentrations are also associated with the emergence of drug‐resistant viruses. Despite increasing advocacy for fair inclusion, in the absence of specific data, concerns about safety resulting from infant exposure during pregnancy and breastfeeding may mean that a woman is offered an older, less well‐tolerated, less effective regimen.^7^

Dolutegravir, an integrase strand transfer inhibitor, was licensed in 2013. WHO guidelines now recommend dolutegravir‐based regimens as first‐line for all individuals, including pregnant women.^8^ An important advantage of dolutegravir is that it reduces the viral load around twice as quickly as other regimens.^9^ This rapid reduction in viral load is also seen when initiated in late pregnancy,^10^ which is an important factor in low‐ and middle‐income countries, where many women present with untreated HIV in late pregnancy.^11^ Dolutegravir is predominately metabolized by UGT1A1 with minor contributions from CYP3A4.^12^ Functional polymorphisms in the UGT1A1 gene that result in decreased enzyme activity for *6, *28, *37 alleles have been shown to affect the dolutegravir exposure in non‐pregnant populations.^13^ The clearance of UGT1A1 PMs was 32% lower than in UGT1A1 EMs in non‐pregnant healthy volunteers. It is uncertain how UGT1A1 genetic polymorphisms, together with the physiological alterations during pregnancy and lactation influence drug exposure to the fetus and exclusively breastfed infants. Therefore, understanding the variability in pharmacokinetics (PK) of dolutegravir in pregnant and lactating mothers, placental transfer, and in infants carrying different UGT1A1 phenotypes, may facilitate optimal dosing of dolutegravir.

Physiologically‐based pharmacokinetic (PBPK) modeling, which accounts for both physiological parameters and drug‐specific characteristics, can be used to supplement available clinical PK data to guide dosing in populations that are otherwise challenging or impossible to evaluate,^14^ such as pregnant women, their fetus or breastfed infant. Both fetal–maternal and pediatric PBPK models incorporate the time varied physiological parameters such as ontogeny of specific enzymes and transporters, which may affect drug disposition under different trimesters for pregnant women and different age groups for pediatrics.^15^, ^16^ In this study, a PBPK model for dolutegravir was developed and verified using available clinical data. The model was then applied to predict the dolutegravir exposure in clinically untested scenarios, namely, different combinations of maternal and neonate/infant UGT1A1 phenotypes. This work aimed to use PBPK predictions to support the safe and effective use of dolutegravir in mothers living with HIV and their babies.

## MATERIALS AND METHODS

### General Workflow

**FIGURE 1:**
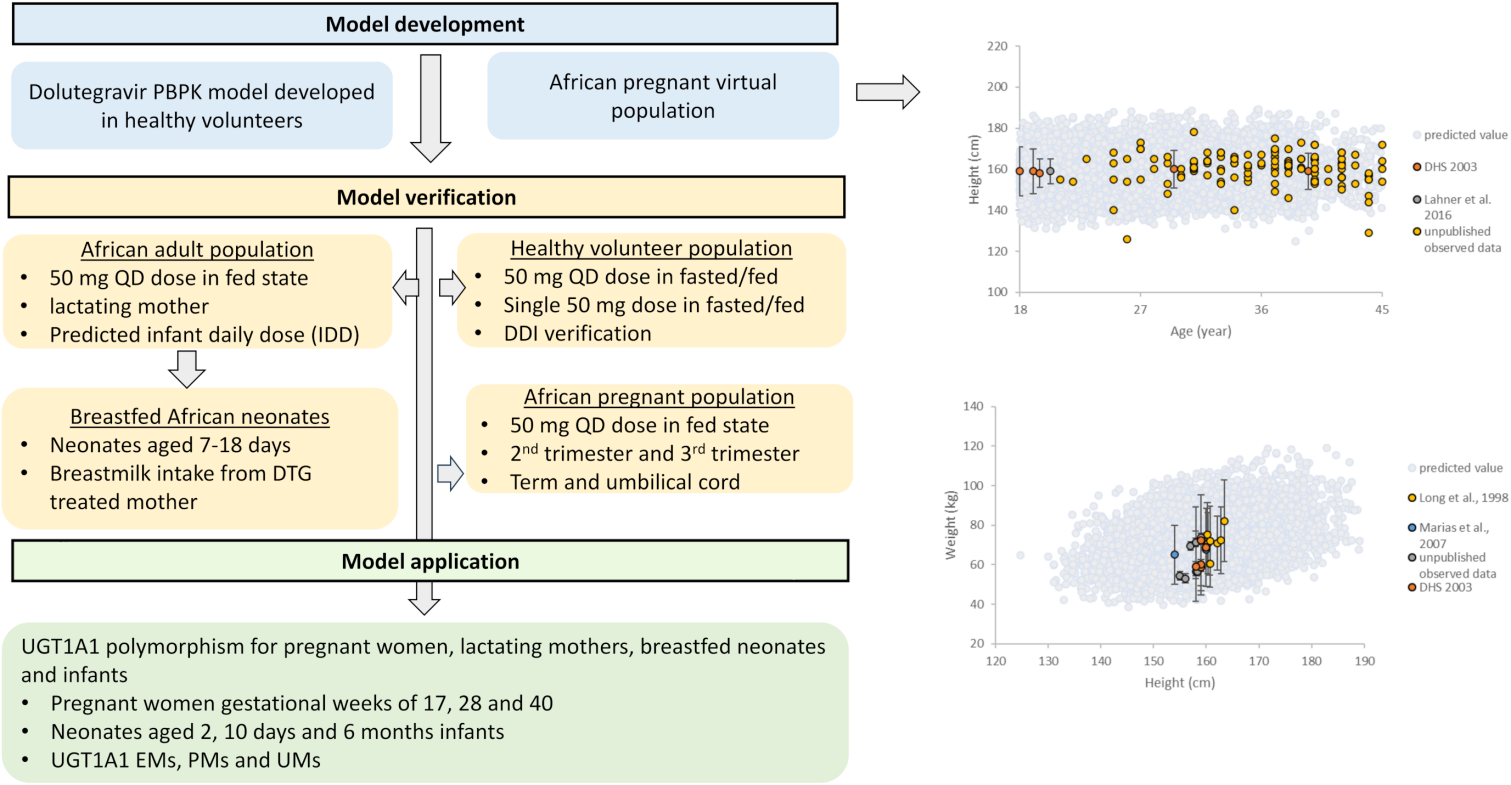
Workflow of the model development, verification, and application of dolutegravir exposure in the studied populations. The panel on the right‐hand side shows the verification of height–age and weight–height relationships for the baseline of Black African pregnant population. The colored data points represent the observed data.^47^, ^48^, ^49^, ^50^ The gray circles are simulated data in 100,000 virtual Black African pregnant at the baseline.

The Simcyp population‐based PBPK simulator (Version 22) was used to predict plasma concentration–time profiles of dolutegravir in pregnant women, umbilical cord, lactating mothers, and breastfed neonates (from birth to 28 days)/infants (28 days–12 months). The workflow of the PBPK model implementation is shown in Figure 1. Firstly, an adult dolutegravir PBPK model was developed and verified in non‐pregnant subjects. Secondly, the model was used to predict the dolutegravir PK during pregnancy using a virtual pregnant population in which the gestational‐dependent changes in the physiological parameters of the mother and the fetus were incorporated. Thirdly, the adult PBPK model was coupled with a lactation model to predict the dolutegravir exposure in maternal plasma, breastmilk and to predict infant daily dose. The predicted infant daily dose from the lactation model was then used as the dose input for the pediatric PBPK model by accounting for age‐dependent physiology changes to predict exposure in breastfed neonates. As the clinical study used to verify the predictions of dolutegravir exposure in pregnant women, lactating mothers, breast milk, and breastfed neonates consisted of subjects living in South Africa and Uganda, corresponding virtual Black African populations were used. The baseline physiological parameters representing non‐pregnant Black African women, including height, weight, body surface area, phenotypic differences for population frequency, enzyme abundances, and cardiac output are adapted from previously published literature.^17^ The maternal age distribution at conception for a total of 1072 subjects in South Africa was collected from the literature.^18^ The virtual pregnancy population accounts for the interindividual variability in physiological parameters during the whole gestational period. The model considers the continuous change of all physiological and biological parameters simultaneously over time.^19^

After demonstrating reasonable recovery of observed PK data for pregnant women, lactating mothers (plasma and breastmilk), and breastfed neonates, the verified models were then used to simulate dolutegravir exposure in UGT1A1 EM, PM, and UM subjects within each of the respective groups. The impact of maternal phenotype on fetal transfer was also simulated.

### Dolutegravir model development and verification

The input parameters for the dolutegravir PBPK model are provided in Table S1. In the current work, a mechanistic oral absorption model was integrated to capture the positive food effect of dolutegravir. The fm of UGT1A1 and CYP3A4 were estimated to be 0.51 and 0.21, respectively.^12^ The remaining 28% was assigned as undefined liver metabolism. The verification of dolutegravir PK prediction in a non‐pregnant population and the fm of both enzymes using available clinical DDI and pharmacogenetic studies (Tables S2 and S3).

### Dolutegravir PK in pregnant women and placental transfer

Although the pregnancy‐related physiological changes incorporated within the model have been described previously,^19^, ^20^ we describe here how UGT1A1 and CYP3A4 alter with gestational week as a key determinant of dolutegravir PK in pregnancy:

$$\mathrm{UGT}1A1={\mathrm{UGT}}_{\left(0\right)}*\left(1+0.01*\mathrm{GW}+0.0002*{\mathrm{GW}}^{2}\right)$$

$$\mathrm{CYP}3A4=\mathrm{CYP}3A4_{\left(0\right)}*\left(1+0.0129*\mathrm{GW}+0.0005*{\mathrm{GW}}^{2}\right)$$

where UGT1A1_(0)_ and CYP3A4_(0)_ are the baseline values in non‐pregnant women and GW is the gestational week. This CYP3A4 equation was derived using dextromethorphan/3‐hydroxymorphinan metabolic ratio and midazolam clearance data after correcting observed data for the binding protein. The UGT1A1 gestational‐related equations were derived based on observed raltegravir clearance in pregnancy.^21^

The fetal model is a multicompartment model coupled with the maternal placenta model described by three compartments.^22^ Equations describing longitudinal changes in fetal and placental physiological parameter values were all incorporated into the model.^23^

Data from an ex vivo placental perfusion experiment^24^ were integrated within the fetal–maternal PBPK model to parameterize the dolutegravir transplacental passage. The reported dolutegravir transplacental transfer was 0.0618 L/h/cotyledon for the perfused cotyledon. The in vitro value was scaled to give a CL_PD_ value of 0.00155 L/h/mL placenta using the equation below:

$${\mathrm{CL}}_{\mathrm{PD}}\;\left(L/h/\mathrm{mL}\;\text{placenta}\right)=\frac{\text{CLcot}\;\left(L/h/\text{cotyledon}\right)*\text{number of cotyledon}}{\text{placenta volume}\;\left(L\right)*1000}$$

where the placenta volume of 0.677 L and the number of cotyledons (17) were applied.

The cord‐to‐maternal plasma ratio was calculated using the equation below:

$$\text{Cord}/{\text{maternal}}_{\text{plasma}}={\mathrm{AUC}}_{\text{umbilical vein plasma}}/{\mathrm{AUC}}_{\text{maternal plasma}}$$

The full list of the virtual trial designs used for pregnant model predictions is provided in Table S4.

### Dolutegravir PK in lactating mothers and breastfed neonates/infants

The integrated lactation model described by Fleishaker, Desai^25^ and Atkinson and Begg^26^ within the Simcyp Simulator V22 was used to predict the milk concentration and milk‐to‐plasma (M/P) ratio for dolutegravir. Due to an absence of reported information on the milk composition in the clinical studies, the physiochemical properties of drug and built‐in mean mature milk properties in the simulator were used. The lactation model has been described in detail elsewhere,^27^ which shows the equations for calculating M/P ratio and concentration in milk. M/P is applied to the dynamic changes in plasma concentration^28^, ^29^ to get concentration–time profiles in milk. Due to the absence of information on the milk properties of the studied lactating mothers, a sensitivity analysis was conducted to evaluate the impact of milk pH and percentage of fat content (% fat) on the predicted M/P.

For simulations in lactating mothers, the virtual Black African population was used assuming physiological changes during pregnancy had returned to pre‐pregnancy baseline values.

The predicted dolutegravir average (*C*_average_ method) and maximum (*C*_max_ method) concentrations from the milk profiles were used to calculate the infant daily dose (IDD) and relative infant daily dose (RIDD) assuming a daily milk intake of 0.15 L/kg in the following equations:

$$\begin{array}{c} \mathrm{IDD}\;\left(\mathrm{mg}/\mathrm{kg}/\mathrm{day}\right)\left(C_{\text{average}}\text{method}\right)\\ \;=\text{average concentration in milk}\;\left(C_{\text{average}};\mathrm{mg}/L\right)\\ \;*\text{daily milk intake}\;\left(L/\mathrm{kg}/\mathrm{day}\right) \end{array}$$

$$\begin{array}{c} \mathrm{IDD}\;\left(\mathrm{mg}/\mathrm{kg}/\mathrm{day}\right)\left(C_{\max}\text{method}\right)\\ \;=\text{maximal concentration in milk}\;\left(C_{\max};\mathrm{mg}/L\right)\\ \;*\text{daily milk intake}\;\left(L/\mathrm{kg}/\mathrm{day}\right) \end{array}$$

$$\text{RIDD}\left(\%\right)=\frac{\mathrm{IDD}\;\left(\mathrm{mg}/\mathrm{kg}/\mathrm{day}\right)}{\text{mother daily dose}\;\left(\mathrm{mg}/\mathrm{kg}/\mathrm{day}\right)}*100$$

where the mother's daily dose is the maternal weight‐adjusted dose.

For assessing the breastfed infant exposure to milk, the predicted IDD was used as the dose input assuming 6 feeds daily for the pediatric PBPK model, which incorporates age‐dependent changes in physiology including UGT1A1 and CYP3A4 (profile 1) using ontogeny equations below:

$$\text{Fraction of adult}=F_{\text{birth}}+\frac{\left(F_{\max}-F_{\text{birth}}\right)*{\mathrm{age}}^{n}}{{\mathrm{Age}}_{50}^{n}+{\mathrm{Age}}^{n}}$$

where for CYP3A4 ontogeny,^30^ *F*_max_ = 1.06, *F*_birth_ = 0.11, Age_50_ = 0.64, *n* = 1.91; for UGT1A1 ontogeny, *F*_max_ = 1, *F*_birth_ = 0, Age_50_ = 0.183, *n* = 1.105.

The full list of the virtual trial designs used for lactation model predictions is provided in Table S4.

### Impact of UGT1A1 phenotype on dolutegravir exposure in the studied populations

After demonstrating reasonable recovery (predicted PK parameters fell within 0.5‐ to 2‐fold of the observed data) of clinical data for pregnant mothers/fetus and lactating mothers/breastfed neonate populations, the verified model was applied to predict dolutegravir exposure in the virtual Black African populations with different UGT phenotypes. The virtual pregnant population with UGT1A1 EMs, PMs, and UMs phenotypes were simulated following 50 mg q.d. dolutegravir orally over 14 days in fed state at gestational weeks of 17, 28, and 40 to predict exposure in pregnant mothers and the placental transfer. The exposure in breastfed neonates (2 and 10 days) and 6‐month‐old infants in UGT1A1 EMs, PMs, and UMs were simulated using predicted IDD (assuming 6 feeds daily) as dose input generated from UGT1A1 EM, PM, and UM breastfeeding mothers, respectively. The full list of the virtual trial designs used for model applications is provided in Table S4.

### Assessment criteria

Dependent on data availability, the predicted PK profiles and/or PK parameters were compared with different sets of clinical observations available in the literature. The PBPK model predictions were considered successful and acceptable if the observed PK profile fell within the 95th and 5th percentile of predicted data and the predicted PK parameters fell within 0.5‐ to 2‐fold of the observed data.

## RESULTS

### Pharmacokinetics of dolutegravir in pregnant women and placental transfer

The PK performance for dolutegravir in non‐pregnant subjects after single and multiple 50 mg q.d. in fasted and fed states is given in Table S2. The prediction errors for *C*_max_, AUC_inf/tau_ and *T*_max_ were within 1.6‐fold of the observed corresponding clinical data parameters. The UGT1A1 and CYP3A4 fms were verified by simulating DDI with inducers (nevirapine, rifampicin, efavirenz, carbamazepine, and rifabutin) and inhibitors (ritonavir and atazanavir) and the results are given in Table S3. Except DDI with carbamazepine and efavirenz, the prediction errors for AUC and *C*_max_ ratio were all within 0.80–1.25‐fold of the observed data.

**FIGURE 2:**
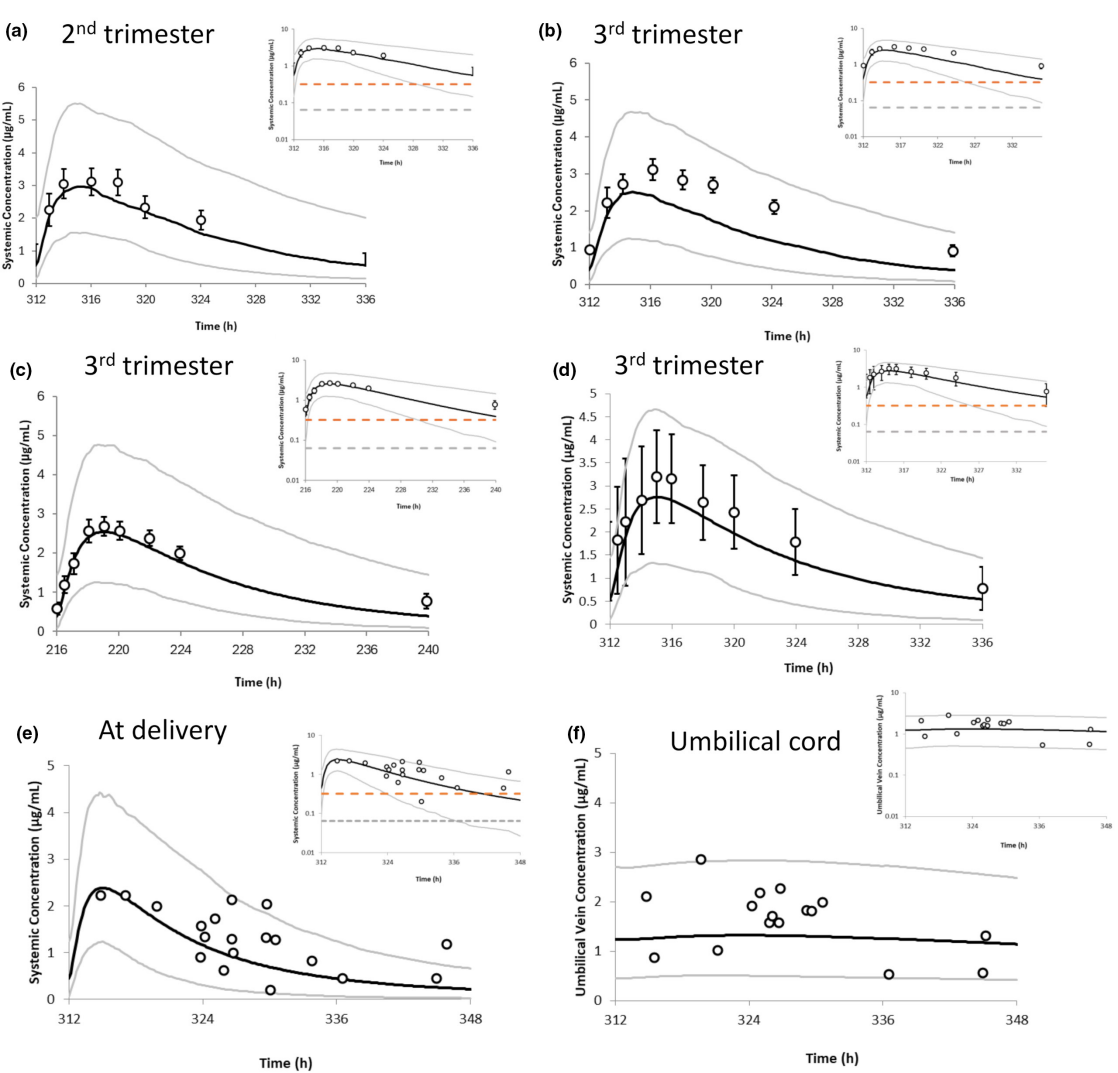
Plasma and umbilical cord concentration–time profiles of dolutegravir following oral administration of 50 mg once a day in fed state in pregnant women, semi‐log scale figures are given as an inset figure in the top‐right corners. The gray and orange dotted lines in the semi‐log figures represent in vitro 90% inhibitory concentration (0.064 μg/mL) and in vivo 90% effective concentration (0.32 μg/mL). The solid line represents the predicted mean/median/geometric mean concentration–time profile, and the gray lines represent the predicted 5th–95th percentile range; (a) Plasma concentration–time profiles of dolutegravir in pregnant women in the second trimester. Empty circles represent in vivo study from Mulligan, Best^32^; (b) Plasma concentration–time profiles of dolutegravir in pregnant women in the third trimester. Empty circles represent in vivo study from Mulligan, Best^32^; (c) Plasma concentration–time profiles of dolutegravir in pregnant women in the third trimester. Empty circles represent the in vivo study from Waitt, Orrell^10^; (d) Plasma concentration–time profiles of dolutegravir in pregnant women in the third trimester. Empty circles represent the in vivo study from Bollen, Freriksen^33^; (e) Plasma concentration–time profiles of dolutegravir with an average gestational age of 38 weeks at delivery. Empty circles represent observed individual data points from Mulligan, Best^32^; (f) Dolutegravir umbilical cord concentration with an average gestational age of 38 weeks at delivery; Empty circles represent observed individual data points from Mulligan, Best.^32^

The predicted dolutegravir exposure during pregnancy is given in Figure 2 for maternal and umbilical cord plasma level. The dolutegravir plasma concentrations were compared with the drug's in vitro IC_90_ of 0.064 μg/mL.^31^ Maternal and fetal concentration predictions were predicted and compared with those available from clinical studies (Table S5). Simulated PK parameters in pregnant subjects were within 1.5‐fold of observed data. The predicted cord‐to‐maternal ratio (at a gestational week of 38) of 1.30 (range: 0.69–2.39) was reasonably consistent with observed ratios of 1.21 (range: 0.51–2.11)^10^ and 1.25.^32^ The predicted AUC of dolutegravir in the second trimester was 58.8% and in the third trimester was 46% of the AUC compared with that of 10 weeks postpartum mothers; corresponding observed differences were 63% for the second trimester and 71% for the third trimester.^32^

### Pharmacokinetics of dolutegravir in lactating mothers and breastfed neonates

**FIGURE 3:**
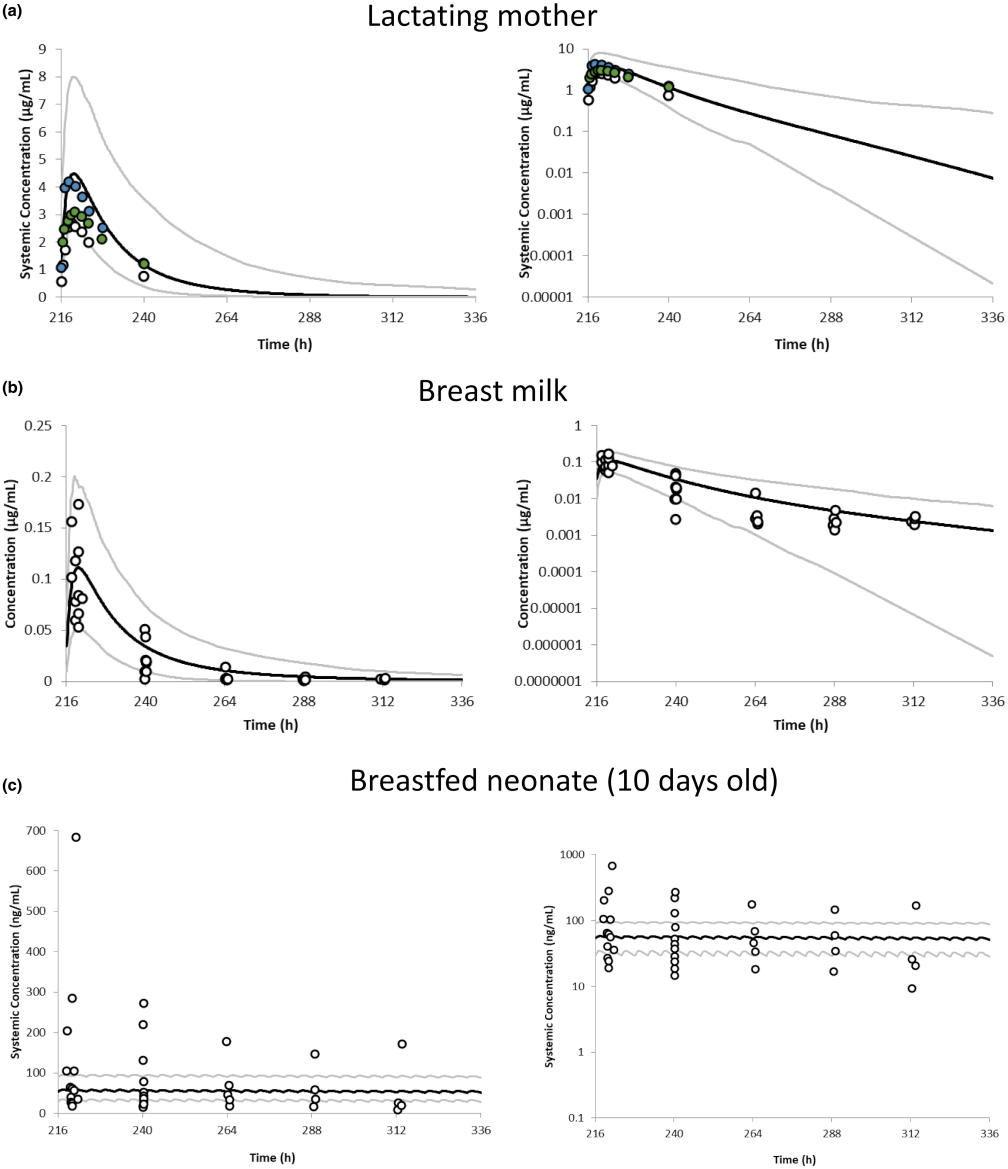
(a) Predicted plasma concentration–time profiles of dolutegravir in Black African postpartum mothers following 50 mg q.d. of dolutegravir in fed state; open circles represent observed PK data in postpartum 1.43 weeks from Waitt, Orrell^10^; green circles represent observed PK data in postpartum 5.9 weeks from Bollen, Freriksen.^33^ Blue circles represent observed PK data in postpartum 10 weeks from Mulligan, Best.^32^ (b) Predicted milk concentration–time profiles of dolutegravir in postpartum 1.43 weeks following 50 mg q.d. of dolutegravir in fed state. Empty circles represent observed individual milk data points in lactating mothers from Waitt, Orrell.^10^ (c) Predicted plasma concentration–time profiles of dolutegravir in breastfed neonates (median 10 days, range 7–18 days) using *C*_average_ for IDD calculation. Empty circles represent observed individual data points in breastfed neonates from Waitt, Orrell.^10^ The solid line represents the predicted mean concentration–time profile and the gray lines represent the predicted 5th–95th percentile range.

The predicted mean plasma dolutegravir concentration–time profile in the virtual Black African female population following daily dosing of 50 mg dolutegravir is reasonably consistent with the observed PK profiles reported in the literature for postpartum mothers^10^, ^32^, ^33^ (Figure 3a). The simulated PK parameters were all within 1.7‐fold of the observed data (Table S5). The predicted dolutegravir concentrations in milk were also in good agreement with observations (Figure 3b) and the lactation‐related parameters are shown in Table S6. The predicted maximal concentration in milk was 1.23‐fold of the observed value. The predicted M/P ratio of 0.023 (range: 0.014–0.045) was in good agreement with the observed value M/P of 0.03 (range 0.03–0.04, *N* = 17)^10^ and M/P of 0.02 (*N* = 1).^34^ The impact of milk pH and %fat on predicted M/P was investigated and the results showed that the %fat has more impact on the predicted M/P compared with milk pH (Table S7).

The predicted IDD and corresponding RIDD, assuming a daily milk intake of 0.15 L/kg, were 0.008 mg/kg/day and 1.5% respectively, using milk *C*_average_ method. The *C*_max_ method‐derived IDD and RIDD were 0.016 mg/kg/day and 3.06%, respectively which can be considered as the “worst case scenario” for neonate exposure from the breast milk.

The pediatric PBPK model was then used to predict the neonatal systemic exposure of dolutegravir after oral ingestion of 0.008 mg/kg/day obtained using *C*_average_ method which was divided equally into 6 daily oral doses to resemble a frequency pattern of breastfeeding after birth, that is, dose was 0.0013 mg/kg at 4 h interval dosing. Figure 3c showed that the predicted mean plasma dolutegravir– time profile in the virtual Black African neonate population following predicted dolutegravir exposure from the milk as the dose input is reasonably consistent with the observed PK profiles.^10^ The predicted *C*_max_ in breastfed neonate plasma was 55.1 ng/mL (range: 21–125 ng/mL) which is consistent with the observed value of 66.7 ng/mL (range: 21–651 ng/mL) as shown in Table S6.

### Impact of UGT1A1 phenotype on dolutegravir exposure in the pregnant populations

**FIGURE 4:**
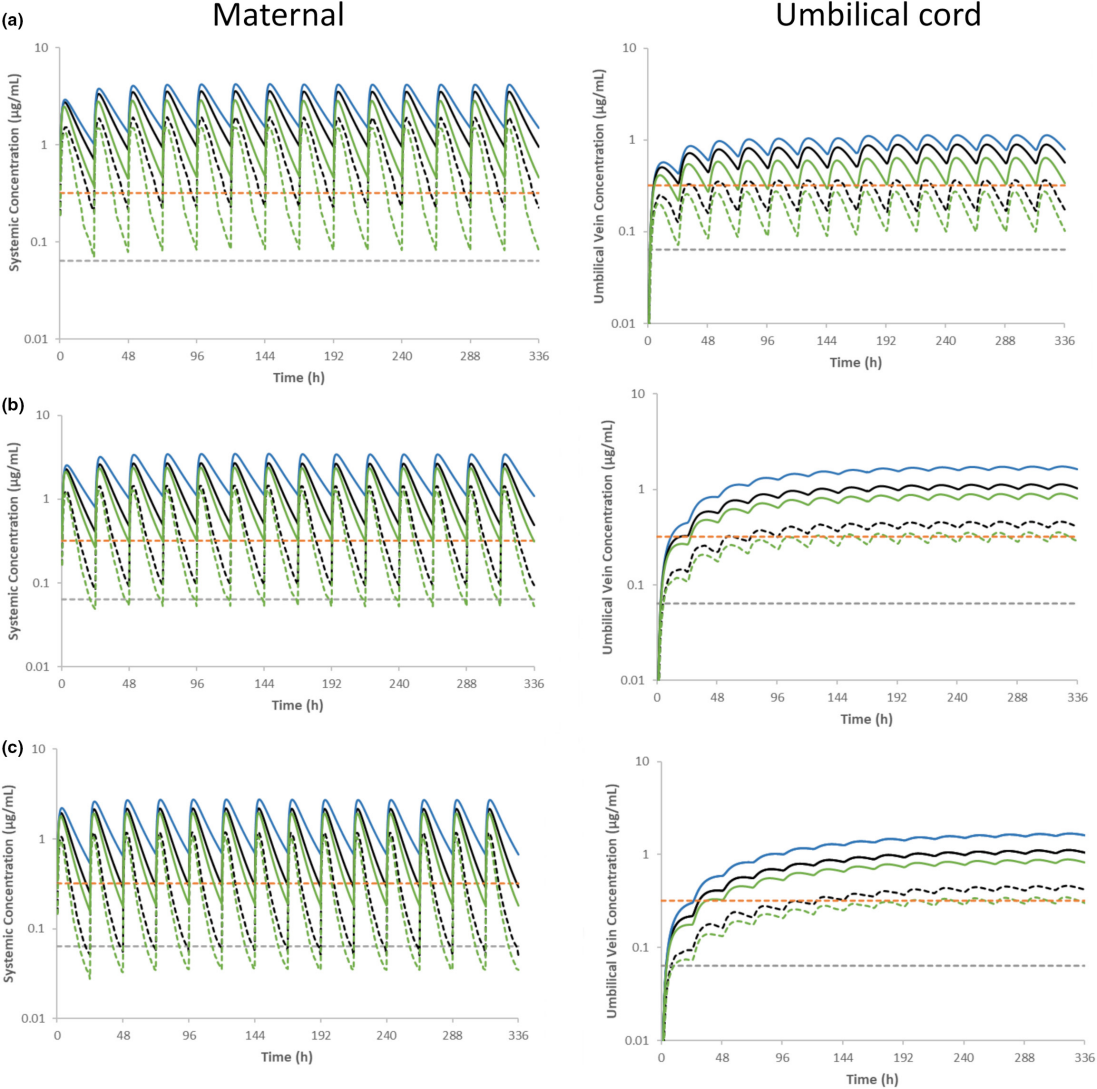
Plasma and umbilical cord concentration–time profiles of dolutegravir following oral administration of 50 mg once a day in fed state in pregnant women with gestational weeks of 17 (a), gestational weeks of 28 (b), and gestational weeks of 40 (c); Blue lines represent predicted concentration–time profile in UGT1A1 PMs; Black lines represent predicted concentration–time profile in UGT1A1 EMs; Green lines represent predicted concentration–time profile in UGT1A1 UMs; the black dash lines represent predicted concentration–time profile at 5th percentile in UGT1A1 EMs; the green dash lines represent predicted concentration–time profile at 5th percentile in UGT1A1 UMs; the gray dash lines represent in vitro 90% inhibitory concentration (0.064 μg/mL); the orange dash lines represent in vivo 90% effective concentration (0.32 μg/mL).

The simulated PK profiles of dolutegravir in UGT1A1 EM, PM, and UM pregnant mothers following 50 mg q.d. dolutegravir orally over 14 days at gestational weeks of 17, 28 and 40 were shown in Figure 4. The dolutegravir plasma concentrations were compared with the drug's in vitro IC_90_ of 0.064 μg/mL, showing that the predicted *C*_min_ dolutegravir concentrations in 95% of UGT1A1 EMs population in gestational weeks 17 and 28 were 3.5‐fold and 1.5‐fold, respectively higher than dolutegravir IC_90_ for both maternal and umbilical cord. At 40 gestational weeks, the predicted *C*_min_ for 5th percentile in UGT1A1 EMs was lower than dolutegravir IC_90_. The predicted mean (±95th percentiles) *C*_min_ for dolutegravir in UGT1A1 UMs population in gestational week 17 was 1.3‐fold higher than dolutegravir IC_90_ for both maternal and umbilical cord. At 28 and 40 gestational weeks, the predicted mean in UGT1A1 UMs was above than that in vitro dolutegravir IC90 although the 5th percentile for *C*_min_ fell below this value. Predicted dolutegravir PK parameters in UGT1A1 EMs, PMs, and UMs (Table 1) showed that the AUC of dolutegravir in both maternal plasma and umbilical cord at GW 17, 28 and 40 in UGT1A1 PMs were about 1.5‐fold and 2.0‐fold higher than those in UGT1A1 EMs and UMs, respectively. The predicted cord‐to‐maternal ratio in UGT 1A1 PMs is comparable to UGT1A1 EMs and UMs at gestational weeks of 17, 28, and 40. For maternal exposure at 40 gestational weeks, the AUC_tau_ was half of the predicted AUC_tau_ at gestational weeks of 17. For transplacental exposure, the cord/maternal ratio was threefold higher and AUC_tau_ in umbilical cord was 1.5‐fold higher at 40 GW compared with those at 17 GW.

**TABLE 1:** Predicted PK parameters in maternal plasma and umbilical cord at the gestational weeks of 17, 28 and 40 in UGT1A1 EMs, PMs and UMs.

Gestational weeks	UGT1A1 genotype	Maternal		Umbilical cord		
		AUC_tau_ (μg/mL.h)	*C*_max_ (μg/mL)	AUC_tau_ (μg/mL.h)	*C*_max_ (μg/mL)	Cord/maternal ratio
17	EM	44	3.3	16	0.80	0.35
	PM	58	3.9	21	1.0	0.35
	UM	30	2.7	11	0.58	0.35
	PM/EM	1.3	1.2	1.3	1.3	1.0
	EM/UM	1.5	1.2	1.5	1.4	1.0
28	EM	30	2.5	23	1.0	0.77
	PM	47	3.2	36	1.5	0.77
	UM	24	2.2	18	0.8	0.77
	PM/EM	1.5	1.3	1.5	1.5	1.0
	EM/UM	1.3	1.1	1.3	1.3	1.0
40	EM	22	2.1	23	1.0	1.1
	PM	34	2.6	36	1.5	1.0
	UM	17	1.8	18	0.79	1.1
	PM/EM	1.6	1.3	1.5	1.5	0.99
	EM/UM	1.3	1.2	1.3	1.3	1.0

### Impact of UGT1A1 phenotype on dolutegravir exposure in lactating mothers

**FIGURE 5:**
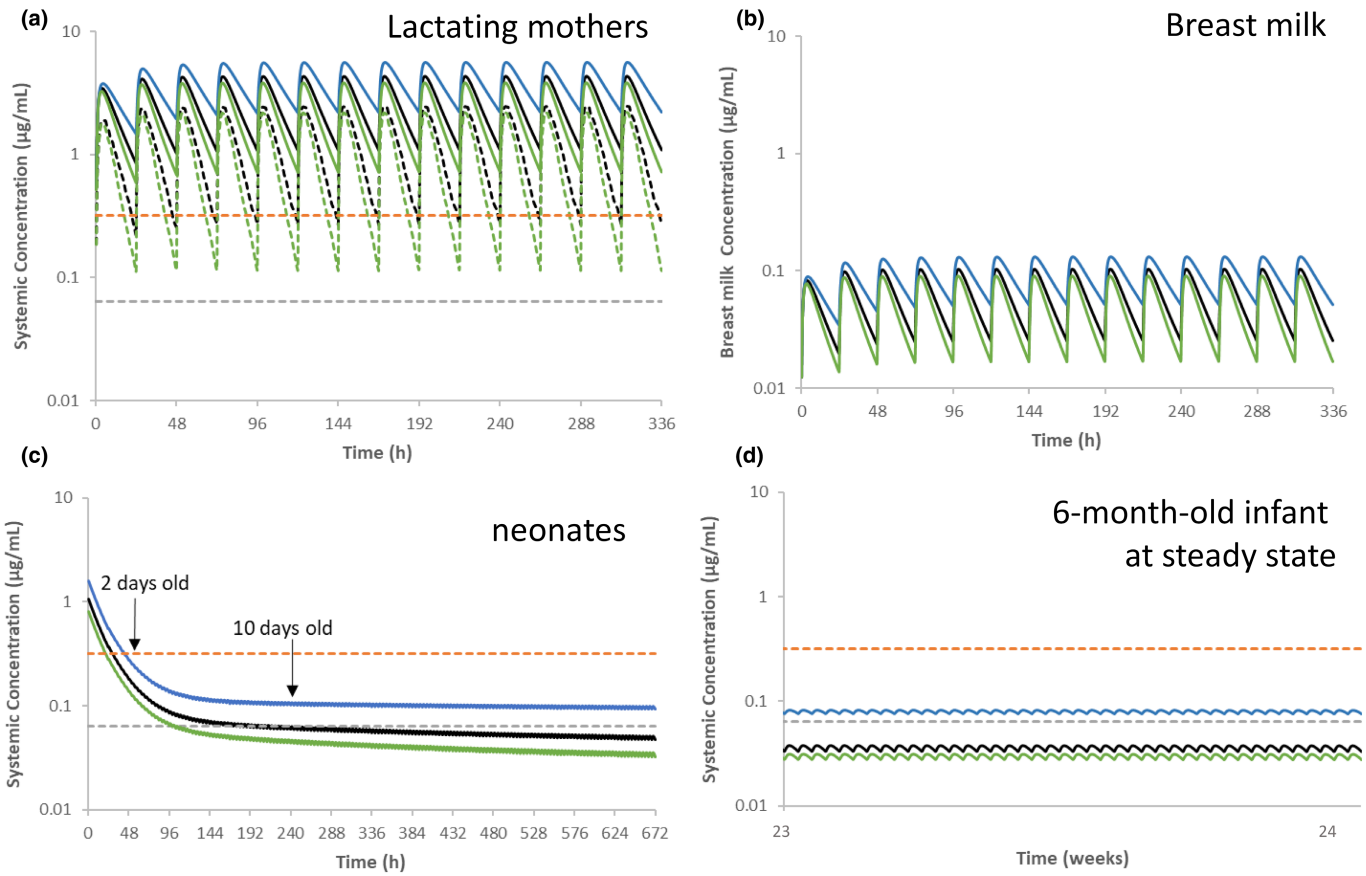
(a) Plasma concentration–time profiles of dolutegravir following oral administration of 50 mg once a day in fed state in lactating mothers; (b) Breast milk concentration–time profiles of dolutegravir following oral administration of 50 mg once a day in fed state in lactating mothers; (c) Plasma concentration–time profiles of dolutegravir over 28 days after delivery. Oral administration of predicted infant daily dose (using milk *C*_average_ method) is simulated considering the cord level as neonate exposure at birth; (d) Plasma concentration–time profiles of dolutegravir at steady state following oral administration (breastfeeding) of predicted infant daily dose using milk *C*_average_ method for 6 months infants; blue lines represent predicted concentration–time profile in UGT1A1 PMs; black lines represent predicted concentration–time profile in UGT1A1 EMs; Green lines represent predicted concentration–time profile in UGT1A1 UMs; the black dash lines represent predicted concentration–time profile at 5th percentile in UGT1A1 EMs; the green dash lines represent predicted concentration–time profile at 5th percentile in UGT1A1 UMs; the gray dash lines represent in vitro 90% inhibitory concentration (0.064 μg/mL); the orange dash lines represent in vivo 90% effective concentration (0.32 μg/mL).

The simulated PK profiles of dolutegravir in lactating mothers, breast milk, breastfed neonates (2 days and 10 days post‐delivery), and infants (6 months post‐delivery) in UGT1A1 EMs, PMs, and UMs following oral administration of 50 mg once a day in fed state in lactating mothers are shown in Figure 5. The dolutegravir plasma concentrations were compared with the drug's in vitro IC_90_ of 0.064 μg/mL showing that the predicted dolutegravir concentrations in 95% of UGT1A1 UMs and EMs population in lactating mothers were above dolutegravir IC_90_. Table 2 presents the predicted IDD and RIDD using *C*_average_ method, and PK parameters of dolutegravir in neonates and after 6 months in breastfed infants in UGT1A1 EMs, PMs, and UMs. The dolutegravir plasma concentrations in breastfed neonates/infants were compared with the drug's in vitro IC_90_ of 0.064 μg/mL showing that the predicted dolutegravir concentrations in 2 days neonates were above dolutegravir IC_90_ with cord level as the baseline, regardless of UGT 1A1 phenotypes. However, for 10‐day‐old breastfed neonates and 6‐months infants, the predicted plasma concentrations for UGT1A1 EMs and UMs were lower than the IC_90_. For lactation parameters, the predicted IDD and RIDD in UGT1A1 PMs were both 1.4‐fold and 1.6‐fold higher than those parameters in UGT1A1 EMs and UMs, respectively. The predicted RIDD in UGT1A1 EMs, PMs, and UMs were lower than the 10% cutoff. The predicted AUC_24h_ in breastfed 2 day‐old neonates, 10 day‐old neonates, and 6 month‐old infants were 1.5‐, 1.7‐, and 2.2‐fold higher in UGT1A1 PMs (with PM mothers) compared with EMs (with EM mothers) but remained 11‐, 41‐, and 72‐fold lower than that observed in the lactating mothers, respectively. Compared with EMs (with EM mothers), the predicted AUC_24h_ in breastfed 2‐day‐old neonates, 10‐day‐old neonates, and 6‐month‐old infants were 1.3‐, 1.3‐, and 1.2‐fold lower in UGT1A1 UMs (with UM mothers) but remained 11–, 42‐ and 68‐fold lower than that observed in the lactating mothers, respectively.

**TABLE 2:** The predicted PK parameters of dolutegravir in lactating mothers, infant daily dose (IDD), and relative infant daily dose (RIDD) using *C*_average_ method and PK parameters in breastfed babies (2 days, 10 days and 6 months post‐delivery) in UGT1A1 EMs, PMs and UMs following 50 mg q.d. dolutegravir in lactating mothers.

UGT1A1 genotype	Lactating mothers		Breast milk		Neonates (2 days old)		Neonates (10 days old)		Infants (6 months old)	
	AUC_24h_ (μg/mL.h)	*C*_max_ (μg/mL)	Infant daily dose (IDD) (*C*_average_ method) (mg/kg/day)	Relative infant daily dose (RIDD) (*C*_average_ method) (%)	AUC_24h_ (μg/mL.h)	*C*_max_ (μg/mL)	AUC_24h_ (μg/mL.h)	*C*_max_ (μg/mL)	AUC_24h_ (μg/mL.h)	*C*_max_ (μg/mL)
EM	58	4.2	0.009	1.3	5.5	0.34	1.4	0.062	0.81	0.036
PM	87	5.4	0.013	1.8	8.4	0.51	2.4	0.100	1.8	0.077
UM	46	3.7	0.008	1.1	4.2	0.26	1.1	0.046	0.68	0.030
PM/EM	1.5	1.3	1.4	1.4	1.5	1.5	1.7	1.6	2.2	2.1
EM/UM	1.3	1.1	1.1	1.2	1.3	1.3	1.3	1.3	1.2	1.2

## DISCUSSION

A PBPK model for dolutegravir was developed and verified before it was applied to predict exposure of dolutegravir in pregnant women, umbilical cord blood, lactating mothers, and breastfed neonates/infants with different combinations of UGT1A1 phenotype. In brief, the fm UGT1A1 (51%) and CYP3A4 (21%) defined in the model, based on in vitro data, were verified using DDI data in non‐pregnant who were predominantly healthy, White subjects. Although several dolutegravir PBPK models have been published previously,^24^, ^35^, ^36^, ^37^ this is the first work studying the impact of UGT1A1 phenotypes on dolutegravir exposure in both pregnant and lactating mothers.

As the majority of studies investigating dolutegravir PK in pregnancy were conducted in Black African subjects, key demographic and physiological parameters defined in the Black African model^17^ were combined with those of the pregnant model to generate a Black African pregnant population, which is more likely to reflect the characteristics of the subjects recruited into the clinical studies. The same approach can be applied to other ethnic groups and verified with clinical PK data. Using this approach, the baseline demographic and body size information for the population of interest is captured and propagated to other physiological parameters in the virtual subjects. Table S8 showed the predicted demographic values and PK parameters of dolutegravir in the Caucasian and Black African pregnant women and indicated ethnicity had minimal impact on the dolutegravir exposure as reported by other investigations.^38^ Accounting for changes in UGT1A1 expression during pregnancy^21^ that have been successfully applied to predict raltegravir (another UGT1A1 substrate) PK in pregnancy,^39^ simulations were in reasonable agreement with the observed (within 1.5‐fold), although slight underprediction was noted. Using data from ex vivo human placenta perfusion experiments^24^ to parameterize the fetal–maternal model, the simulated cord‐to‐maternal ratio of 1.3 was in good agreement with clinical observations (within 1.1‐fold).

Once verified, the model was then applied to prospectively predict the impact of UGT1A1 phenotype on maternal and transplacental exposure. The predicted mean total systemic and umbilical vein concentrations were in excess of the dolutegravir IC_90_ for wild‐type virus at 17, 28, and 40 gestational weeks, regardless of UGT1A1 phenotype. However, at 40 gestational weeks, the 5th percentile of maternal concentrations for UGT1A1 EMs and UMs indicated trough exposure was 1.3‐fold and 1.8‐fold lower than the IC_90_ in vitro, respectively. These simulations suggest that the standard dose of dolutegravir (50 mg q.d.) in fed state is generally appropriate in late pregnancy, regardless of UGT1A1 phenotype. As a significant proportion of women are diagnosed late in pregnancy in sub‐Saharan Africa, with as much as 20% initiating antiretroviral therapy in the third trimester,^11^ these simulations supplement the available clinical data and offer reassurance around the use of standard dosing regimens. It is important to note an in vivo EC_90_ ^31^, ^40^ that is significantly higher than the reported in vitro IC_90_.^31^ In this analysis, we have indicated both concentrations on figures for comparison but have used the in vitro value to infer adequacy of exposure. This is due to limitations in the clinical study where a PK/PD relationship could not be derived^40^ and in keeping with recent publications from the DolPHIN‐1 study.^10^, ^41^

For simulations in lactating women, it was assumed that physiological changes during pregnancy had returned to pre‐pregnancy baseline values. With this assumption, the maternal dolutegravir concentrations in 10 weeks postpartum women were well recovered,^32^ although those at 1.4‐ and 5.9‐week postpartum were somewhat over‐predicted (within 1.7‐fold),^10^, ^33^ as observed exposure at these timepoints were more in line with those in pregnancy. Potentially, this may be due to the oversimplification of transition from the pregnant to non‐pregnant state, particularly in the early days postpartum, supported by recent data showing that adaptation postpartum may be prolonged.^42^ Despite this, predictions were still in reasonable agreement and the impact of this assumption is equal across UGT1A1 phenotypes. Hence, the model was deemed fit‐for‐purpose.

Using the Fleishaker/Atkinson and Begg model,^25^, ^26^ the predicted dolutegravir M/P ratio was consistent with those observed clinically. In the absence of milk composition and pH data from clinical studies, default values for mature milk (pH 7; %fat = 3.9%) were applied. Assuming a standard milk consumption of 150 mL/kg/day indicated an IDD of 0.016 mg/kg/day and an RIDD much less than 10%, the cutoff is generally used as an indication of safety,^43^ in line with clinical data. When this dose was split across 6 equal feeds and the median infant age (10 days) from the clinical study was used in simulations, the model was able to accurately recover the central tendency neonate exposure (with 0.83‐fold of observed *C*_max_) but underestimated the variability and upper range of observed. In the absence of information detailing the age of each individual infant, the neonate exposure resulting from transplacental exposure was assumed to be negligible. However, the upper range of clinical observations could be more accurately captured if a younger age was simulated where the initial “dose” from placental transfer was not completely washed out. Figure S1 shows the plasma concentration–time profiles of dolutegravir over 7 days after delivery in UGT1A1 EM and PM neonates considering the cord concentration as neonate exposure at birth. This figure shows that the upper range and trend of clinical observations could be more accurately captured if assuming neonate plasma was sampled at 2 days postpartum plasma sampling and UGT1A1 PM phenotype was assumed. Clearly, these are assumptions but this exercise highlights the sensitive parameters in the model and their importance for consideration in prospective predictions for neonates of this age.

Despite progress in the reduction of MTCT of HIV worldwide, transmission through breastfeeding still contributes to almost 50% of pediatric HIV infections recorded every year.^44^ Although the chances of transmission are greatly reduced when the mother is virologically suppressed, there are cases of transmission when the mother had an undetectable viral load.^45^ Although simulations indicated that even the exposure in breastfed infants who were UGT1A1 PMs was unlikely to reach toxic levels, consideration of dolutegravir exposure in breastfed neonates and infants in the context of prophylactic coverage or the potential to select for viral resistance, and the identification of covariates such as UGT1A1 phenotype, may be important. Therefore, the model was applied to assess the role of UGT1A1 phenotype differences in breastfed neonates (2 and 10 days old) and infants (6 months old), respectively, a 1.5‐, 1.7‐, and a 2.2‐fold difference in exposure was predicted between UGT1A1 PMs and EMs. The UGT1A1 phenotype differences between EM and UM were 1.3‐, 1.3‐, and 1.2‐fold in breastfed neonates (2 and 10 days old) and infants (6 months old), respectively. The predicted mean concentrations in 2‐day‐old neonates were in excess of the dolutegravir IC_90_ assuming cord concentrations (from EM, PM, and UM mothers) as baseline, regardless of UGT1A1 phenotype. However, for 10‐day‐old breastfed neonates and 6‐month‐old infants, predicted concentrations for UGT1A1 EMs and UMs were lower than the IC_90_. These results indicate that the potential prophylactic coverage generally remained for 2 days post‐birth due to transplacental transfer of dolutegravir and the immaturity of dolutegravir elimination mechanisms at birth. Overall, breastfeeding contributed relatively little to infant plasma exposure, and the prophylactic coverage waned with time as umbilical cord concentration were cleared.

A caveat to this work is the assumption of static breastmilk characteristics when pH and % fat change as breastmilk matures.^46^ To assess the potential impact of alterations in breastmilk characteristics, especially since we are simulating neonate exposure, sensitivity analyses were carried out to assess the impact of changes in milk characteristics to those more typical of colostrum compared with mature milk and indicated minimal impact on the M/P ratio across that range (Table S7). Human milk fat concentration is highly variable and increases with longer breastfeeding duration and also increases during each breastfeeding moment.^46^ Extension of the sensitivity analysis to cover a broader range of pH and % fat indicated that % fat has more impact on predicted M/P and IDD compared with pH. When the % fat is up to 6%, the predicted M/P and IDD using *C*_average_ method was 1.3‐fold and 1.4‐fold, respectively higher than the corresponding values predicted using default milk properties (pH = 7, % fat = 3.9%). It is worth noting that the exposure in breastfed infants who are UGT1A1 PMs still has predicted %RIDD <10% when the % fat is 6%.

In summary, we report the verification and application of a dolutegravir PBPK model to supplement clinical data and predict the impact of UGT1A1 phenotype on dolutegravir exposure in pregnant women placental transfer and breastfed neonates/infants. These simulations allow the assessment of maternal and pediatric UGT1A1 phenotype to further support the safe and effective use of the standard 50 mg q.d. regimen of dolutegravir in pregnant and lactating women.

## AUTHOR CONTRIBUTIONS

J.N and L.M.A. wrote the manuscript. L.M.A., J.N., A.P., K.R.Y., A.T.H., and C.W. designed the research. J.N. and L.M.A. performed the research. L.M.A. and J.N. analyzed the data.

## FUNDING INFORMATION

This work was funded by the Bill and Melinda Gates Foundation (INV‐040110). The views expressed in this work do not reflect the official views of the Bill & Melinda Gates Foundation. CW is funded by the Wellcome Trust Clinical Research Career Development Fellowship (222075_Z_20_Z).

## CONFLICT OF INTEREST STATEMENT

L.M.A., J.N., A.P., K.R.‐Y., A.T.H. are employees of Certara Predicted Technologies and may hold shares in Certara. All other authors had no competing interests to declare. As Deputy Editor in Chief of CPT: Pharmacometrics & Systems Pharmacology, Karen Rowland Yeo was not involved in the review or decision process for this paper.

## Supporting information


Data S1
