# Supplementary material for: Using PBPK modeling to supplement clinical data and support the safe and effective use of dolutegravir in pregnant and lactating women
Source: CPT Pharmacometrics Syst Pharmacol. 2024 Oct 30;13(11):1924–38. doi: 10.1002/psp4.13251 (PMC11578129; doi:10.1002/psp4.13251)
Supplement: Supplementary file 1 — Data S1 [file PSP4-13-1924-s001.docx]

Using PBPK modeling to supplement clinical data and support safe and effective use of dolutegravir in pregnant and lactating women

Jia Ning^1^, Amita Pansari^1^, Karen Rowland Yeo^1^, Aki T. Heikkinen^1^, Catriona Waitt^2,3,4^ and Lisa M. Almond^1^^

^1^Certara Predicted Technologies, Sheffield, United Kingdom

^2^Department of Clinical Pharmacology and Therapeutics, University of Liverpool, Liverpool, United Kingdom

^3^Infectious Disease Institute, Makerere University College of Health Sciences, Kampala, Uganda

^4^Royal Liverpool University Hospital, Liverpool, United Kingdom

^corresponding author

Table S1 Input parameters used for the dolutegravir PBPK model

| **Parameter** | **Value** | **Note** |
| --- | --- | --- |
| MW | 419.38 | NDA ^1^ |
| log P | 2.16 | NDA ^1^ |
| Compound type | Monoprotic acid |  |
| pKa | 8.2 | TGA ^2^ |
| B:P | 0.55 | NDA ^1^ reported B/P range of 0.44-0.54 |
| fu | 0.006 | NDA ^1^ and Moss, Wagner ^3^ |
| Binding protein | HSA | NDA ^1^ |
| Absorption | ADAM, solution with precipitation | |
| Peff (10-4cm/s) | 2.93 | Predicted from Papp of MDCKII cells, calibrated against propranolol ^1^ |
| fu_gut_ | 0.14 | Predicted with MDCKII cells |
| Solubility (mg/mL) | 0.019 | pH at 1.2 from NDA ^1^ |
| SF1 | 14.2 | Estimated in SIVA4 using pH dependent solubility data from NDA ^1^ |
| Bile micelle mediated solubility | LogKm:w neutral: 3.95  LogKm:w Ion: 6.08 | Estimated in SIVA4 using biorelevant media (FaSSIF and FeSSIF) solubility data from NDA ^1^ |
| Critical Supersaturation Ratio | 2 | Optimized value to pick up fa for fasted state |
| DLM scaler | 0.4 | Optimized value to pick up fa for fasted state |
| Particle size (uM) | Polydisperse | Based on D10, D50 and D90 reported from Alves, de Marchi ^4^ |
| Distribution | Full PBPK, method 3, Kp scaler 1 | Reported Vss is not available, use of Song, Borland ^5^ fasted PK profile to evaluate the distribution |
| CYP3A4  (µl/min/pmol CYP3A4) | 0.0463 | Retrograde from observed fasted CLpo data, assuming fa of fasted state is 0.54, CYP3A4 fm of 21% and UGT1A1 fm of 51% are reported by Reese, Savina ^6^ |
| UGT1A1 (µl/min/mg protein) | 16.94 |  |
| Additional HLM (µl/min/mg protein) (undefined liver metabolism) | 7.545 |  |
| OCT2 Ki (µM) | 0.39* | The lowest in vitro measured value in HEK cells from Tátrai, Schweigler ^7^ |
| Fumic | 1 | default |
| MATE Ki (µM) | 0.3* | Optimized value (10-fold reduction) from the lowest in vitro measured value in HEK cells from Saito, Ishiguro ^8^ |
| Fumic | 1 | default |

MW= molecular weight; LogP=neutral species octanol: buffer partition coefficient; pKa= acid dissociation constant; B/P=blood-to-plasma partition ratio; fup=fraction unbound in plasma; ADAM= Advanced Dissolution, Absorption and Metabolism; Peff = Effective human jejunum permeability; fu_gut_ = fraction of drug unbound in the gut, i.e. within the enterocyte; SF=solubility factor; CSR=critical supersaturation ratio; DLM= diffusion layer model; V_ss_=volume of distribution at steady state; Kp=Tissue-plasma partition coefficient; CYP = cytochrome P450;fm= Fraction of drug metabolised; fu_mic_= fraction unbound in *in vitro* microsomal incubation; HLM=human liver microsome. *Due to changes in the operational concentrations in subsequent versions of the Simulator, these dolutegravir values are modified in Version 23

Table S2 Comparison of predicted and observed pharmacokinetic parameters in non-pregnant population.

| clinical study | dose regimen | AUC_tau_/_inf_ (µg.h/ml) | | | C_max_ (µg/ml) | | | T_max_ (h) | | |
| --- | --- | --- | --- | --- | --- | --- | --- | --- | --- | --- |
|  |  | predicted | observed | pre/obs | predicted | observed | pre/obs | predicted | observed | pre/obs |
| Dumitrescu, Peddiraju ^9^* | single 50 mg oral fasted | 52.3  (26.2) | 60.9  (20.1) | **0.86** | 1.96  (0.62) | 3.27  (1.05) | **0.60** | 2.41  (0.86-6.05) | 1.50  (0.751-3.00) | **1.61** |
| Dumitrescu, Peddiraju ^9^* | single 50 mg oral fed | 75.2  (37.1) | 78.2  (17.3) | **0.96** | 3.36  (1.19) | 3.83  (0.80) | **0.88** | 4.21  (1.80-10.98) | 5.00  (1.01-8.00) | **0.84** |
| Song, Borland ^10^* | single 50 mg oral fasted | 62.9  (30) | 40.3  (15.1) | **1.56** | 2.07  (0.61) | 1.97  (0.86) | **1.05** | 2.50  (0.83-5.65) | 3.00  (1.0-4.0) | **0.83** |
| Song, Borland ^5^^ | single 50 mg oral fed | 69.3  (49) | 71.0  (31) | **0.98** | 3.27  (36) | 4.03  (19) | **0.81** | 4.18  (1.78-10.9) | 4.00  (2.0-6.0) | **1.05** |
| Ross, Song ^11^^ | 50 mg qd for 5 days oral fasted | 37.5  (43) | 35.7  (34.7) | **1.05** | 2.57  (32) | 2.65  (34.7) | **0.97** | 1.98  (0.65-3.95) | NA | **NA** |
| Johnson, Borland ^12#^ | 50 mg qd for 5 days oral fed | 65.6  (63.04-68.2) | 68.9  (60.3-78.6) | **0.95** | 4.34  (4.19-4.49) | 4.99  (4.43-5.62) | **0.87** | 3.75  (1.90-7.65) | 3.00  (1.0-4.0) | **1.25** |

* arithmetic mean (SD) except median T_max_ (range)

^ geometric mean (CV%) except median T_max_ (range)

^#^ geometric mean (90% confidence interval) except median T_max_ (range)

Table S3 Comparison of predicted and observed DDI studies when dolutegravir is a substrate or perpetrator in non-pregnant population.

| clinical study | dose regimen | | Geometric mean AUC ratio (90% CI) | | | Geometric mean Cmax ratio (90% CI) | | |
| --- | --- | --- | --- | --- | --- | --- | --- | --- |
|  | substrate | perpetrator | predicted | observed | pre/obs | predicted | observed | pre/obs |
| Dailly, Allavena ^13^* | 50 mg qd dolutegravir for 5 days in fed state | **nevirapine** 400 mg qd for 5 days | 0.78  (0.77-0.80) | 0.81  (0.69-0.94) | 0.96 | 0.87  (0.86-0.88) | 0.92  (0.83-1.02) | 0.95 |
| Dooley, Sayre ^14^ | 50 mg qd dolutegravir on day 1-7, 50 mg bid on day 8-28 in fasted state | **rifampicin** 600 mg qd on day 15-day 28 | 0.42  (0.40-0.43) | 0.46  (0.38-0.55) | 0.91 | 0.54  (0.53-0.56) | 0.57  (0.49-0.65) | 0.95 |
| Dooley, Sayre ^14^ | 50 mg qd dolutegravir on day 1-day 21 in fasted state | **rifabutin** 300 mg qd on day 8-day 21 | 0.88  (0.87-0.89) | 0.95  (0.82-1.10) | 0.93 | 0.93  (0.92-0.93) | 1.16  (0.98-1.37) | 0.80 |
| Song, Borland ^15^ | 30 mg qd dolutegravir for 14 days in fed state | **ritonavir** 100mg qd together with **atazanavir** 300 mg qd for 14 days | 1.51  (1.48-1.53) | 1.62  (1.50-1.74) | 0.93 | 1.29  (1.27-1.31) | 1.34  (1.25-1.42) | 0.96 |
| Song, Borland ^15^ | 30 mg qd dolutegravir for 14 days in fed state | **atazanavir**  400 mg qd for 14 days | 1.53  (1.50-1.56) | 1.91  (1.80-2.03) | 0.80 | 1.31  (1.29-1.33) | 1.5  (1.50-1.59) | 0.87 |
| Song, Borland ^16^ | 50 mg qd dolutegravir for 14 days in fasted state | **efavirenz** 600 mg qd for 14 days | 0.66  (0.65-0.68) | 0.43  (0.35-0.54) | 1.53 | 0.81  (0.80-0.82) | 0.61  (0.51-0.73) | 1.33 |
| Song, Weller ^17^ | 50 mg qd dolutegravir in fed state on day 1-day 5 | **carbamazepine** 300 mg bid on day 1-day 5 | 0.87  (0.86-0.88) | 0.51  (0.48-0.55) | 1.71 | 0.92  (0.92-0.93) | 0.67  (0.61-0.73) | 1.37 |
| Wang, Cerrone ^18^ | 50 mg qd dolutegravir in fed state on day 1-day 7 at 9 am | **rifampicin** 600 mg qd on day 1-day 7 at 8:30 am | 0.46  (0.44-0.47) | 0.44  (0.37-0.52) | 1.05 | 0.68  (0.66-0.69) | 0.65  (0.55-0.75) | 1.05 |
| Wang, Cerrone ^18^ | 50 mg qd dolutegravir in fed state on day 1-day 7, 100 mg qd on day 8-day 14 at 9 am in fed state | **rifampicin** 600 mg qd on day 1-day 14 at 8:30 am | 0.43  (0.42-0.45) | 0.42  (0.35-0.50) | 1.02 | 0.63  (0.62-0.64) | 0.64  (0.55-0.74) | 0.98 |
| Song, Zong ^19^ | 500 mg bid metformin on day 1-day 7 | **dolutegravir** 50 mg bid on day 1-day 7 | 2.45  (2.25-2.66) | 2.09  (2.02-2.16) | 0.85 | 2.11  (1.91-2.33) | 1.70  (1.66-1.75) | 0.81 |

*geometric mean (95% CI)

Table S4 Virtual trial settings for pregnant women, lactating mothers and UGT1A1 phenotype on dolutegravir exposure in the studied populations

| The following trial designs were set for model prediction to match the clinical studies |
| --- |
| **Pregnant women** |
| Trial design P1: multiple oral doses of 50 mg QD of dolutegravir at steady state ^20^; 10 trials of 15 black pregnant women aged 21-42 at median 23.9 gestational weeks. |
| Trial design P2: multiple oral doses of 50 mg QD of dolutegravir at steady state ^20^; 10 trials of 28 black pregnant women aged 21-42 at median 33.7 gestational weeks. |
| Trial design P3: multiple oral doses of 50 mg QD of dolutegravir at steady state ^20, 21^; 10 trials of 28 black pregnant women aged 21-42 at delivery (38 gestational weeks). |
| Trial design P4: multiple oral doses of 50 mg QD of dolutegravir at steady state ^22^; 10 trials of 15 black pregnant women aged 21-42 at median 33.4 gestational weeks. |
| Trial design P5: multiple oral doses of 50 mg QD of dolutegravir at steady state ^23^; 10 trials of 29 black pregnant women aged 19-42 at median 31 gestational weeks. |
| **Lactating women** |
| Trial design L1: multiple oral doses of 50 mg QD of dolutegravir at steady state ^22^; 10 trials of 10 black lactating mothers aged 21-42 at median postpartum 5.9 weeks. |
| Trial design L2: multiple oral doses of 50 mg QD of dolutegravir at steady state ^20^; 10 trials of 22 black lactating mothers aged 21-42 at median postpartum 10 weeks. |
| Trial design P3: multiple oral doses of 50 mg QD of dolutegravir at steady state ^23^; 10 trials of 17 black pregnant women aged 19-42 at median postpartum 1.4 weeks (Breast milk PK data available). |
| Trial design N1: multiple oral doses of the predicted average infant daily dose divided into 6 daily doses for 14 consecutive days ^23^; 10 trials of 17 (50% female) black breastfed infants at median 10 days (range 7-18 days) post-delivery |
| **UGT1A1 phenotype on dolutegravir exposure in the studied populations** |
| Trial design AP1: multiple oral doses of 50 mg QD of dolutegravir over 14 days; 10 trials of 10 black pregnant women aged 18-45 at either 17, 28, or 40 GWs in UGT1A1 EMs,PMs or UMs. |
| Trial design AL1: multiple oral doses of 50 mg QD of dolutegravir over 14 days; 10 trials of 10 black lactating mothers aged 18-45 in UGT1A1 EMs, PMs and UMs. |
| Trial design AN1: multiple oral doses of the predicted average infant daily dose from UGT1A1 EM lactating mothers divided into 6 daily doses for 14 consecutive days; 10 trials of 10 (50% female) black UGT1A1 EM breastfed infants at either 2, 10 days or 6-months post-delivery. A single intravenous loading dose of 0.12 mg/kg (for 2 and 10 days neonates) was administered over 10 s to produce an initial systemic concentration of 1 µg/mL, the same concentration as was predicted in the cord plasma at birth. |
| Trial design AN2: multiple oral doses of the predicted average infant daily dose from UGT1A1 PM lactating mothers divided into 6 daily doses for 14 consecutive days; 10 trials of 10 (50% female) black UGT1A1 PM breastfed infants at either 2, 10 days or 6-months post-delivery. A single intravenous loading dose of 0.18 mg/kg (for 2 and 10 days neonates) was administered over 10 s to produce an initial systemic concentration of 1.5 µg/mL, the same concentration as was observed in the cord plasma at birth. |
| Trial design AN3: multiple oral doses of the predicted average infant daily dose from UGT1A1 UM lactating mothers divided into 6 daily doses for 14 consecutive days; 10 trials of 10 (50% female) black UGT1A1 UM breastfed infants at either 2, 10 days or 6-months post-delivery. A single intravenous loading dose of 0.09 mg/kg (for 2 and 10 days neonates) was administered over 10 s to produce an initial systemic concentration of 0.79 µg/mL, the same concentration as was observed in the cord plasma at birth. |

Table S5 Predicted and observed pharmacokinetic parameters of dolutegravir in pregnant and lactating mother.

|  | Geometric mean AUC_tau_ (µg.h/ml) | | | Geometric C_max_ (µg/ml) | | | References |
| --- | --- | --- | --- | --- | --- | --- | --- |
|  | predicted | observed | pre/obs | predicted | observed | pre/obs |  |
| **pregnant women** |  |  |  |  |  |  |  |
| second trimester, gestational weeks=23.9* | 39.6 | 47.6 | 0.83 | 3.03 | 3.62 | 0.84 | Mulligan, Best ^20^ |
| third trimester, gestational weeks=33.7* | 31.0 | 49.2 | 0.63 | 2.57 | 3.54 | 0.72 | Mulligan, Best ^20^ |
| third trimester, gestational weeks=31 | 31.5 | 35.3 | 0.89 | 2.60 | 2.53 | 1.03 | Waitt, Orrell ^23^ |
| third trimester, gestational weeks=33.4 | 31.6 | 40.8 | 0.78 | 2.60 | 3.15 | 0.83 | Bollen, Freriksen ^22^ |
| **lactating mothers** |  |  |  |  |  |  |  |
| postpartum 1.43 weeks | 67.2 | 40.1 | 1.67 | 4.57 | 2.90 | 1.58 | Waitt, Orrell ^23^ |
| postpartum 5.9 weeks | 67.1 | 47.0 | 1.43 | 4.58 | 3.34 | 1.37 | Bollen, Freriksen ^22^ |
| postpartum 10 weeks* | 67.3 | 65.0 | 1.04 | 4.50 | 4.85 | 0.93 | Mulligan, Best ^20^ |

* median PK parameters

Table S6 Predicted and observed dolutegravir lactation parameters

| parameters | predicted  (range) | observed  (range) | pre/obs | references |
| --- | --- | --- | --- | --- |
| Breast milk_max_ (ng/ml) (N=17) | 104  (41.9-316) | 84.6  (43.8-171) | 1.23 | Waitt, Orrell ^23^ |
| Milk/plasma ratio (N=17) | 0.023  (0.014-0.045) | 0.03  (0.03-0.04) | 0.77 | Waitt, Orrell ^23^ |
| Milk/plasma ratio (N=1) | 0.023 | 0.02 | 1.15 | Kobbe, Schalkwijk ^24^ |
| Infant daily dose (mg/kg/day)  (C_av_ method) | 0.008  (0.0031-0.024) | 0.015 | 0.53 | Kobbe, Schalkwijk ^24^  (calculation method is not mentioned) |
| Infant daily dose (mg/kg/day)  (C_max_ method) | 0.016  (0.0063-0.047) | 0.015 | 1.07 | Kobbe, Schalkwijk ^24^  (calculation method is not mentioned) |
| Infant_max_ (ng/ml)  (C_average_ method) | 55.1  (21-125) | 66.7  (21-654) | 0.83 | Waitt, Orrell ^23^ |
| Relative infant daily dose (%)  (C_average_ method) | 1.55 | NA | NA |  |
| Relative infant daily dose (%)  (C_max_ method) | 3.06 | NA | NA |  |
| Infant_max_ (ng/ml)  (C_average_ method) | 55.1  (21-125) | 66.7  (21-654) | 0.83 | Waitt, Orrell ^23^ |

Table S7 Impact of milk pH and %fat on predicted M/P and IDD

| Milk pH | % fat | Predicted M/P (range) | Predicted IDD  (C_av_ method) (range) | Note |
| --- | --- | --- | --- | --- |
| 6.8 | 1% | 0.012 | 0.0040 |  |
|  |  | (0.0082-0.018) | (0.0017-0.011) |  |
| 6.8 | 3.90% | 0.022  (0.012-0.044) | 0.0044  (0.0036-0.029) |  |
| 7 | 3.90% | 0.023 | 0.0079 | Default parameters |
|  |  | (0.014-0.045) | (0.0032-0.024) |  |
| 7.4 | 3.90% | 0.023 | 0.0080 | Colostrum |
|  |  | (0.014-0.045) | (0.0032-0.024) |  |
| 7.4 | 6% | 0.031 | 0.011 |  |
|  |  | (0.018-0.064) | (0.0042-0.033) |  |

Table S8 Predicted physiological parameters and pharmacokinetic parameters of dolutegravir in Caucasian and black African pregnant mother at the third trimester

|  | Body weight (kg) (CV%) | Liver weight (g) (CV%) | AUC_tau_(µg.h/ml) (CV%) | C_max_ (µg.h/ml) (CV%) |
| --- | --- | --- | --- | --- |
| Caucasian | 76.9 (13%) | 1445 (14%) | 34.3 (51%) | 2.75 (40%) |
| Black African | 82.6 (17%) | 1622 (16%) | 31.0 (53%) | 2.57 (44%) |


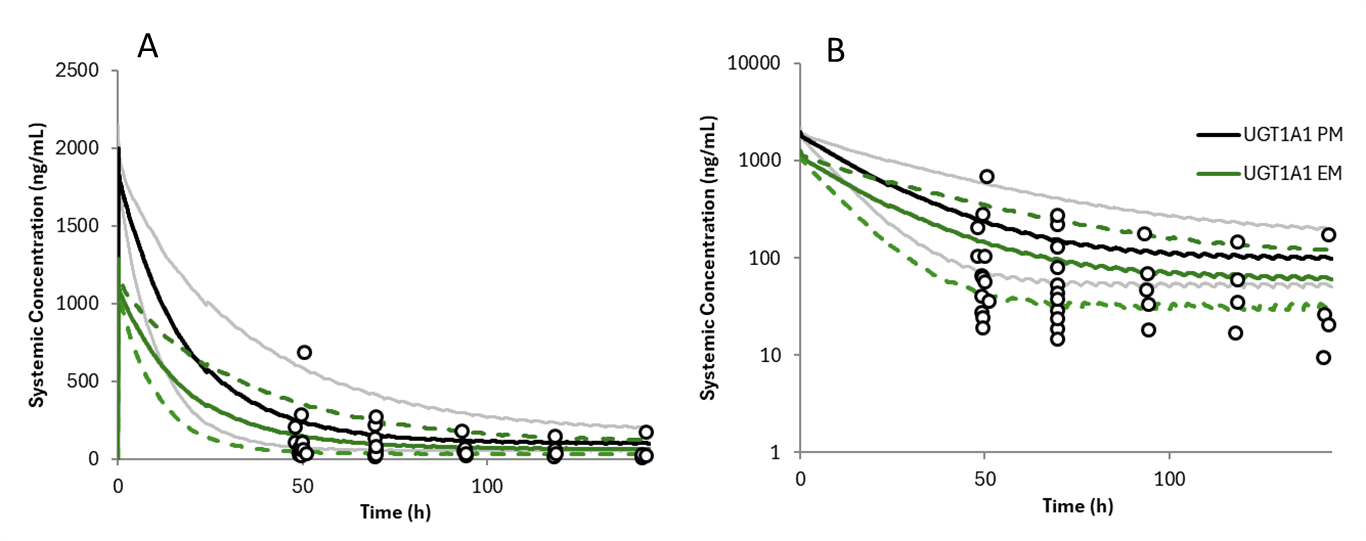


Figure **S1**. Linear (A) and semi-log scale (B) plasma concentration–time profiles of dolutegravir over 7 days after delivery. Oral administration of predicted infant daily dose (using milk C_average_ method) is simulated considering the cord level as neonate exposure at birth in UGT1A1 EM (green line) and PM (black line); The green dash lines and grey lines represent the predicted 5^th^–95^th^ percentile range in UGT1A1 EM and PM, respectively.

**References**

1. NDA. Clinical Pharmacology review 204790.

2. TGA. Product information-TIVICAY (dolutegravir) tablets; 2014.

3. Moss L, Wagner D, Kanaoka E*, et al.* The comparative disposition and metabolism of dolutegravir, a potent HIV-1 integrase inhibitor, in mice, rats, and monkeys. *Xenobiotica* (2015); **45** 60-70.

4. Alves M, de Marchi AA, Doretto KM*, et al.* Searching for a long-acting injectable formulation for the antiretroviral dolutegravir. *British Journal of Pharmacy* (2019); **4** 2.1-2.12.

5. Song I, Borland J, Chen S*, et al.* Effect of food on the pharmacokinetics of the integrase inhibitor dolutegravir. *Antimicrob Agents Chemother* (2012); **56** 1627-1629.

6. Reese MJ, Savina PM, Generaux GT*, et al.* In vitro investigations into the roles of drug transporters and metabolizing enzymes in the disposition and drug interactions of dolutegravir, a HIV integrase inhibitor. *Drug Metab Dispos* (2013); **41** 353-361.

7. Tátrai P, Schweigler P, Poller B*, et al.* A Systematic In Vitro Investigation of the Inhibitor Preincubation Effect on Multiple Classes of Clinically Relevant Transporters. *Drug Metab Dispos* (2019); **47** 768-778.

8. Saito A, Ishiguro N, Takatani M, Bister B, Kusuhara H. Impact of Direction of Transport on the Evaluation of Inhibition Potencies of Multidrug and Toxin Extrusion Protein 1 Inhibitors. *Drug Metab Dispos* (2021); **49** 152-158.

9. Dumitrescu TP, Peddiraju K, Fu C*, et al.* Bioequivalence and Food Effect Assessment of 2 Fixed-Dose Combination Formulations of Dolutegravir and Lamivudine. *Clin Pharmacol Drug Dev* (2020); **9** 189-202.

10. Song IH, Borland J, Savina PM*, et al.* Pharmacokinetics of Single-Dose Dolutegravir in HIV-Seronegative Subjects With Moderate Hepatic Impairment Compared to Healthy Matched Controls. *Clin Pharmacol Drug Dev* (2013); **2** 342-348.

11. Ross LL, Song IH, Arya N*, et al.* No clinically significant pharmacokinetic interactions between dolutegravir and daclatasvir in healthy adult subjects. *BMC Infect Dis* (2016); **16** 347.

12. Johnson M, Borland J, Chen S*, et al.* Effects of boceprevir and telaprevir on the pharmacokinetics of dolutegravir. *Br J Clin Pharmacol* (2014); **78** 1043-1049.

13. Dailly E, Allavena C, Grégoire M*, et al.* Influence of nevirapine administration on the pharmacokinetics of dolutegravir in patients infected with HIV-1. *J Antimicrob Chemother* (2015); **70** 3307-3310.

14. Dooley KE, Sayre P, Borland J*, et al.* Safety, tolerability, and pharmacokinetics of the HIV integrase inhibitor dolutegravir given twice daily with rifampin or once daily with rifabutin: results of a phase 1 study among healthy subjects. *J Acquir Immune Defic Syndr* (2013); **62** 21-27.

15. Song I, Borland J, Chen S*, et al.* Effect of atazanavir and atazanavir/ritonavir on the pharmacokinetics of the next-generation HIV integrase inhibitor, S/GSK1349572. *Br J Clin Pharmacol* (2011); **72** 103-108.

16. Song I, Borland J, Chen S*, et al.* Effects of enzyme inducers efavirenz and tipranavir/ritonavir on the pharmacokinetics of the HIV integrase inhibitor dolutegravir. *Eur J Clin Pharmacol* (2014); **70** 1173-1179.

17. Song I, Weller S, Patel J*, et al.* Effect of carbamazepine on dolutegravir pharmacokinetics and dosing recommendation. *Eur J Clin Pharmacol* (2016 a); **72** 665-670.

18. Wang X, Cerrone M, Ferretti F*, et al.* Pharmacokinetics of dolutegravir 100 mg once daily with rifampicin. *Int J Antimicrob Agents* (2019); **54** 202-206.

19. Song IH, Zong J, Borland J*, et al.* The Effect of Dolutegravir on the Pharmacokinetics of Metformin in Healthy Subjects. *J Acquir Immune Defic Syndr* (2016 b); **72** 400-407.

20. Mulligan N, Best BM, Wang J*, et al.* Dolutegravir pharmacokinetics in pregnant and postpartum women living with HIV. *Aids* (2018); **32** 729-737.

21. Liu XI, Momper JD, Rakhmanina NY*, et al.* Prediction of Maternal and Fetal Pharmacokinetics of Dolutegravir and Raltegravir Using Physiologically Based Pharmacokinetic Modeling. *Clin Pharmacokinet* (2020); **59** 1433-1450.

22. Bollen P, Freriksen J, Konopnicki D*, et al.* The Effect of Pregnancy on the Pharmacokinetics of Total and Unbound Dolutegravir and Its Main Metabolite in Women Living With Human Immunodeficiency Virus. *Clin Infect Dis* (2021); **72** 121-127.

23. Waitt C, Orrell C, Walimbwa S*, et al.* Safety and pharmacokinetics of dolutegravir in pregnant mothers with HIV infection and their neonates: A randomised trial (DolPHIN-1 study). *PLoS Med* (2019); **16** e1002895.

24. Kobbe R, Schalkwijk S, Dunay G*, et al.* Dolutegravir in breast milk and maternal and infant plasma during breastfeeding. *Aids* (2016); **30** 2731-2733.
